# Predation risk affects growth and reproduction of an invasive snail and its lethal effect depends on prey size

**DOI:** 10.1371/journal.pone.0187747

**Published:** 2017-11-14

**Authors:** Jing Guo, Pablo R. Martín, Chunxia Zhang, Jia-en Zhang

**Affiliations:** 1 Department of Ecology, College of Natural Resources and Environment, South China Agricultural University, Guangzhou, China; 2 Laboratorio de Ecología, INBIOSUR (UNS/CONICET), Departmento de Biología, Bioquímica y Farmacia, Universidad Nacional del Sur, Bahía Blanca, Argentina; 3 Key Laboratory of Agro-Environment in the Tropics, Ministry of Agriculture, Guangzhou, China; 4 Guangdong Engineering Research Center for Modern Eco-agriculture and Circular Agriculture, Guangzhou, China; Tokai University, JAPAN

## Abstract

The behavior of invasive species under predation risk has been studied extensively, but their growth and reproductive responses have rarely been investigated. We conducted experiments with juveniles and adults of the invasive freshwater snail *Pomacea canaliculata*, and we observed changes in growth and reproduction in response to predation risk from a caged predator (*Trachemys scripta elegans*). *P*. *canaliculata* produced eggs earlier in the presence of predators and injured conspecifics compared with the control group (no risk), although the total number of egg masses laid by per female was exceeded by that of the controls after 15 days. Egg hatching success noticeably decreased under predation risk, and the incubation period was significantly prolonged; however, the oviposition height of the snails was not affected. A lethal effect of predation risk was detected in juvenile snails but not in adults. The growth of juvenile *P*. *canaliculata* was inhibited under predation risk, probably due to a reduction in food intake. Adult females exhibited a greater reduction in growth under predation risk than males, which likely resulted in part from the high reproductive investment of females in egg laying. These results indicate that *P*. *canaliculata* snails under predation risk face a trade-off between predator avoidance and growth and reproduction, where the lethal effect of predation risk is linked to the size of the prey.

## Introduction

Most organisms must simultaneously consume enough energy for themselves while avoiding becoming prey for predators [1]. They optimize trade-offs among different aspects (survival, growth, reproduction, and so on) with energy limitation under predation risk [2–5]. Ample evidence has indicated that prey growth, reproduction and behavior can be altered after perceived predation risk [2,6–8]. Birth and growth rates are determinant of prey demography. Changes in birth and growth rates under predation risk have attracted theoretical and empirical attention since the end of the last century [3]. Growth is a crucial ecological trait that determines population development, and, in prey, this trait is affected by predation risk [4]. Moreover, an elevated predation risk is associated with reproductive activities (e.g., copulation, pregnancy, spawning and breeding) because prey may exhibit morphological or behavioral changes that make them vulnerable to predators during these activities [5]. The predator-sensitive food hypothesis suggests that prey species often reduce foraging activity or efficiency in response to predation risk, although this behavior can ultimately lead to marked decreases in growth and reproduction [2,6,9,10].

In this study, we assessed the role of predation risk in the growth and reproductive output of the dioecious freshwater snail *Pomacea canaliculata*, which is an invasive species in many areas of the world, particularly in East and Southeast Asia [11,12]. The rapid growth and high reproductive capacity of *P*. *canaliculata* are crucial for its ability to colonize and establish populations in rice fields and other freshwater ecosystems [13,14]; these snails reach sexual maturity in 2–3 months [15] and breed two to three generations per year [16] under ideal natural conditions.

Dozens of potential biological control agents have been studied to control the population growth of *P*. *canaliculata* [17,18], but a few species can be applied to control snails in rice fields to large effect, including the mallard duck *Tadorna tadorna* [19] and the common carp *Cyprinus carpio* [20]. One of the major reasons that the snail *P*. *canaliculata* displays diverse avoidance behaviors is to address the threat from predators [21]. These snails close their operculum tightly to avoid predation [22], crawl above the waterline, or burrow into sediment when they perceive predation risk in the form of infochemicals from predators, artificially crushed conspecifics [23,24] or conspecifics that have been eaten or injured by predators [25,26]. They can recognize predators by associative learning, which might help them face many novel predators, especially as an invasive species [24]. However, the growth and reproductive responses of *P*. *canaliculata* under predation risk are poorly understood, although Ichinose and Tochihara [27] found that under predation risk from the common carp *Cyprinus carpio*, the growth of *P*. *canaliculata* was unaffected and the investment in egg production increased.

In the present study, the red-eared slider turtle *Trachemys scripta elegans* was used to simulate predation risk. This turtle is often kept as a pet in China, but it is an invasive alien species native to the southeastern United States [28]. *P*. *canaliculata* is native to South America [13] and has no co-evolutionary history with this turtle species. In China, most wild populations of these species do not co-exist under natural conditions, though a few populations of these two species co-exist in some habitats, such as ponds and streams.

Under predation risk by caged *T*. *scripta elegans*, the growth of juvenile and adult *P*. *canaliculata* is expected to be inhibited since their avoidance behaviors are performed at the expense of feeding time [22]. We studied their survival rates in the context of the juveniles being more sensitive to predation risk [23]. Moreover, we measured the growth and survival of male and female adult snails separately with regard to the sex differences in their alarm response [29] and reproductive effort. In addition, we investigated the predator-induced reproductive plasticity of *P*. *canaliculata* by assessing the effects of predation risk on the timing of egg mass laying, the number of eggs, oviposition height, egg hatching success, and incubation period. Finally, we assessed the trade-offs made by the invasive snail between predation risk avoidance and growth and reproduction on basis of these studies. These findings should be conducive to a more rational appraisal of the biocontrol efficiency of different predators on *P*. *canaliculata*, since most studies have only considered the direct predation effects imposed on snails by predators [18,30,31].

## Materials and methods

### Ethics statement

All experiments were carried out in adherence to institutional guidelines for animal welfare and were conducted under the protocol approved by the Animal Ethics Committee of the South China Agricultural University (2015–06). We conducted experiments using *P*. *canaliculata* snails collected from an irrigation ditch near rice fields at the teaching and scientific research base of South China Agricultural University in central Guangdong Province, China (23°17’ N, 113°37’ E), where there are no endangered or protected species. No specific permits were required for the field study and the locations are not protected.

### Experimental materials

The collected snails were reared in a 135-L rectangular aquarium (70 cm long × 52 cm wide × 37 cm high) and fed lettuce (*Lactuca sativa*) under conditions of natural sunlight and a temperature of 28±1°C. Mature and juvenile individuals were selected seven days before their use in the experiments. The snails often reach sexual maturity at 25 mm in shell height [25]. The shell height was measured with a Vernier caliper from the shell apex to the extreme basal aperture [32]. No turtle species were found at the collection site during the ten previous years according to our own observations during several studies. Twelve turtles (*T*. *scripta elegans*) weighing approximately 210 g each were purchased from Yuehe Pet Market in Guangzhou (23°09’ N, 113°24’ E) and reared in an outdoor rectangular aquarium with the same size as described above. The aquarium was tilted to ensure that the turtles had enough dry space to rest (approximately one-third of the bottom of the aquarium). The turtles were fed live *P*. *canaliculata*, and the water was changed every three days. The snails supplied *ad libitum* as food were small (<20 mm shell height) so that the turtles could devour them quickly, thus reducing their pain. Rearing lasted for more than 20 days in order to acclimatize the animals to the experimental conditions.

### Experimental procedure

**Fig 1:**
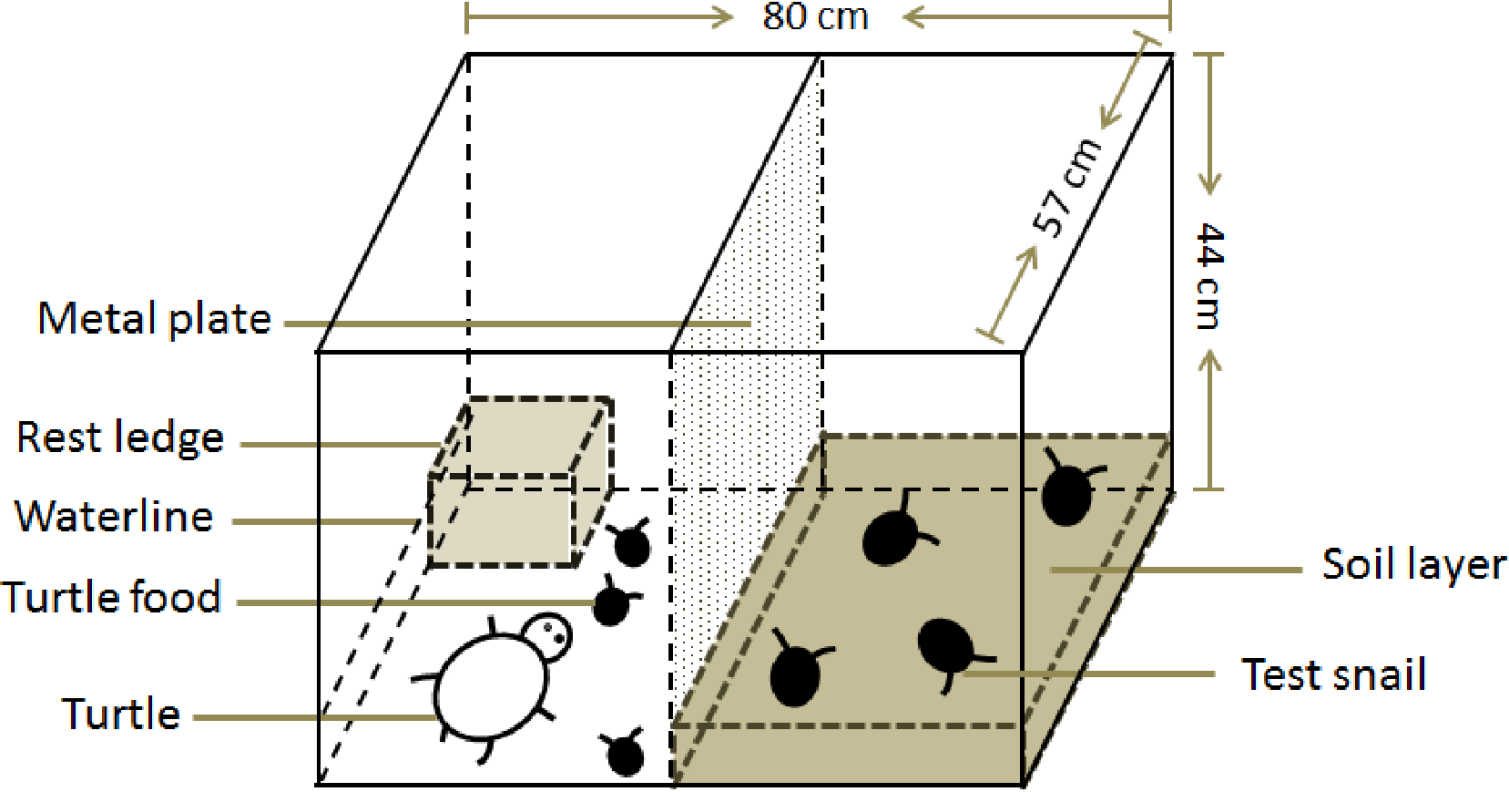
Diagram of the aquarium used in the experiment.

Six 200-L polyethylene aquaria were placed outdoors in natural light and used to survey the growth, reproduction and survival of adult *P*. *canaliculata* under predation risk. The aquaria were divided into two equal compartments by a 0.9-mm thick metal plate with 5-mm circular holes to allow for the passage of chemical signals from turtles and injured snails but to prevent the passage of the turtles and snails from one compartment to the other (Fig 1). In each aquarium, a 5-cm deep layer of sediment was placed at the bottom of one compartment; the distance from the water surface to the top of the sediment was approximately 10 cm. The sediment was obtained from a paddy field, air dried for a week and thoroughly mixed before use. In the other compartment, a plastic box (21.5 cm long × 14 cm wide × 16 cm high) was provided as a resting site for the turtles.

Like juvenile *P*. *canaliculata*, the opercula of females remain concave after sexual maturity, whereas those of adult males become convex in the labral fringe close to the penis sheath [33]; observing this difference is the common method for determining the sex of a snail. Twenty adult snails (10 females and 10 males) with shell heights of 25–31 mm were placed in the compartment that contained sediment, and after 24 h of acclimation, two turtles and several *P*. *canaliculata* snails (10–20 mm in shell height) were placed in the compartment without sediment. Each aquarium was covered with plastic nets (2-mm mesh size) to prevent the snails and turtles from escaping and mixing, and the base of the metal plate was sealed with transparent tape to a height of 5 cm to prevent the soil from entering the other section. The six aquaria were equally divided into two groups: predation risk and no predation risk (control). The predation risk group was as described above, and the design of the control was identical except for the absence of turtles.

The test snails were fed lettuce (*L*. *sativa*) and water lettuce (*Pistia stratiotes*), which were supplied daily. The number of snails provided as turtle food was checked every day and new snails were added in order to maintain a minimum of five live individuals during the experiment. The experiment began when the turtles were introduced. For all of the egg masses laid by the test snails, the date and the height of oviposition (from the lower point of each egg mass to the water surface) were recorded. Although the height of the aquaria used in this experiment was limited so that the maximum oviposition height of *P*. *canaliculata* could not exceed 26 cm, more than 90% of the egg masses did not touch the top. We checked the egg masses and the food provided for the test snails or turtles each morning because oviposition often occurs at night [34,35]. The date of oviposition was recorded as the day prior to finding the egg masses. Approximately 5 cm of the water in the aquaria was replaced with tap water each week to avoid water fouling.

The experiment lasted 30 days and the water temperature fluctuated between 25 and 30°C. The survival rate and growth of the test snails were examined at the end of the experiment; the snail live weight was measured with a digital scale and the weight growth rate of males and females was calculated from the averages of the living snails in each aquarium.

All of the *P*. *canaliculata* egg masses were carefully detached from the aquarium walls and metal plates every two days with ultrathin blades. We randomly selected 4–6 egg masses in each aquarium for transfer indoors; then were incubated at 27±2°C under natural light to determine hatch success. The number of newborn snails and the day of the first hatching in each egg mass were recorded. The incubation period was calculated from the day the eggs were laid to the day of the first hatching [27]. The number of eggs in each of the non-incubated egg masses was counted after the eggs were unfastened from each other in a 2% sodium hydroxide solution for 1.5 h [36]. The total number of eggs in each aquarium was calculated as the sum of the number of eggs in all egg masses.

Six small aquaria (56 cm long × 45 cm wide × 35 cm high) with the same design as described above (Fig 1) were used to determine juvenile snail growth, feeding and survival under predation risk, regardless of sex. The juvenile snails (shell height from 7 to 12 mm) were fed daily with pre-weighed lettuce (*L*. *sativa*) and their intake was assessed by measuring the wet weigh of the surplus lettuce after 24 hours. The experiment lasted 25 days, and the growth and survival of juveniles were estimated using the aforementioned methods. No turtles died during the two experiments, and all of the turtles continued to grow after the tests.

### Data analysis

All of the statistical analyses were performed using SPSS version 20.0 (IBM Inc., USA). Normality of data was tested by using the Shapiro-Wilk test, and the Mann-Whitney U test was used to compare the six aquaria between the control and predation risk groups when this assumption was not met. When the data satisfied the normal distribution, the effect of predation risk on the periodic egg mass production per female was analyzed every three days and cumulative one at the end of the experiment, using an independent sample *t*-test with the aquaria as replicates. The growth and survival of adult and juvenile *P*. *canaliculata*, the total eggs laid by females, and the food intake of juvenile snails under predation risk were also analyzed using independent sample *t*-test. A nested ANOVA was used to analyze the effect of predation risk on the oviposition height, hatching success, incubation period and clutch size (i.e., number of eggs per egg mass) of *P*. *canaliculata*; predation risk was categorized as a main effect, and the aquaria was categorized as a nested effect with each egg mass as a replicate. Differences were considered significant at *p*<0.05. The values are presented as the means with standard errors.

## Results

**Fig 2:**
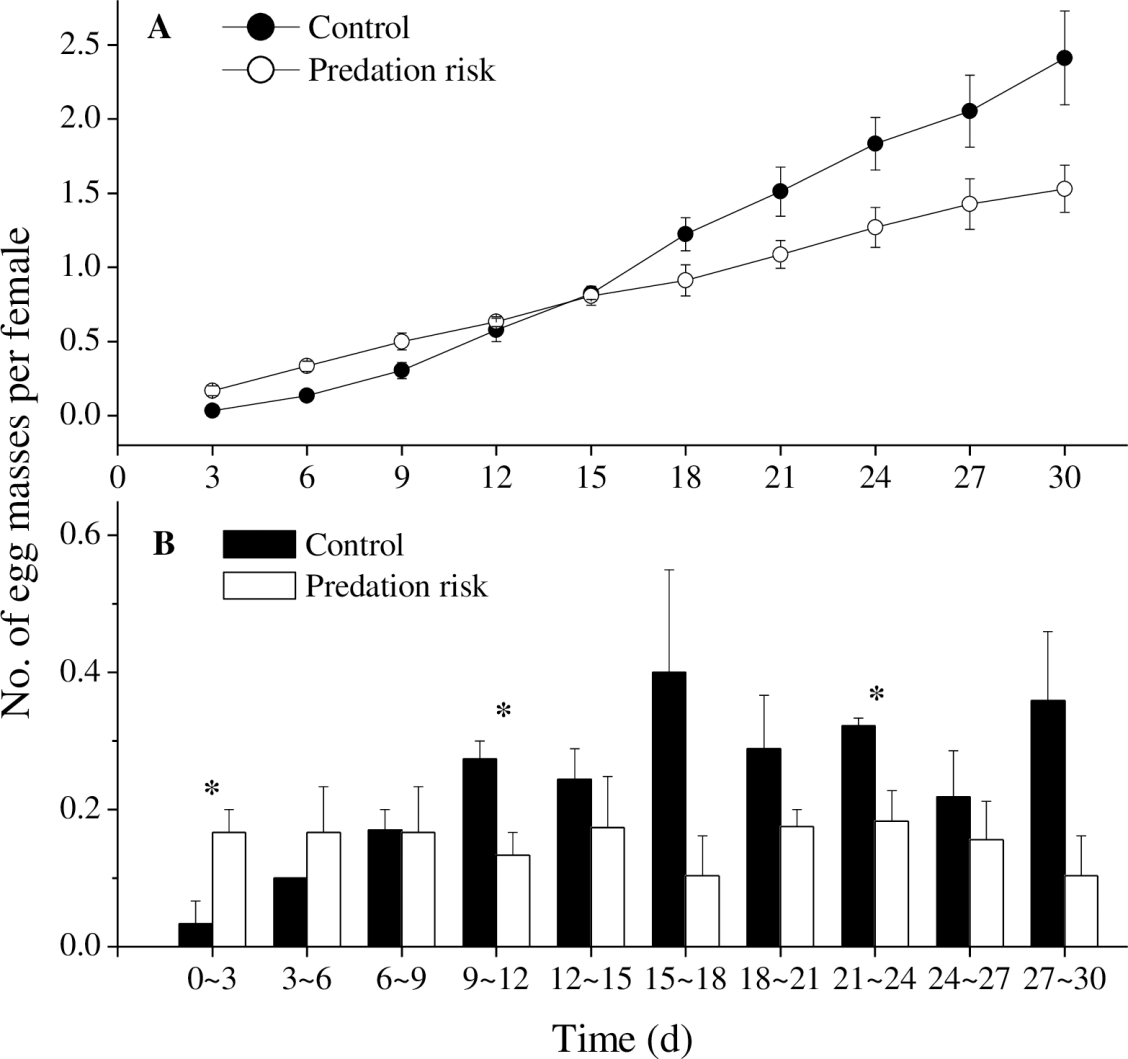
**Cumulative (A) and Periodic (B) Egg Mass Production of *Pomacea canaliculata* under Predation Risk.** The asterisks indicate significant differences in the numbers of egg masses laid by per female between the two groups. * *p*<0.05. Error bars represent standard errors of the mean in panels A and B. *N* = 6.

More than 80 snails per aquarium were eaten by *T*. *scripta elegans* during the two experiments. Over our many observations, approximately half of the adult and juvenile *P*. *canaliculata* often buried themselves under predation risk; holes in the soil surface as well as buried snails were observed frequently in the turtle group but rarely in the control group, indicating that the turtles provided a consistently high degree of predation risk. When adult *P*. *canaliculata* were under predation risk, egg masses were found in two aquaria on the first day; these egg masses were found earlier than in the control, in which one egg mass first emerged on the third day. Moreover, the number of egg masses laid by per female under predation risk was significantly higher than that in the control during the first three days (*t* = -2.828, df = 4, *p* = 0.047, Fig 2B). Subsequently, from the 9th-12th day, the number of egg masses increased in the absence of turtles and surpassed the number of egg masses in the predation risk group (*t* = 3.333, df = 4, *p* = 0.029), and this trend continued to the end of this experiment (Fig 2B). During the second half of the study, the number of cumulative egg masses of *P*. *canaliculata* under predation risk was gradually overtaken by the control. The gap widened as the experiment proceeded, and by the end of the trial, the cumulative number of egg masses laid by per female was 1.53±0.16 in the predation risk group and 2.41±0.32 in the control group, but the difference was not significant (*t* = 2.492, df = 4, *p* = 0.067, Fig 2A).

The clutch sizes were significantly different in the control and predation risk groups, (117.13 and 94.43 eggs, respectively) (Tables 1 and 2). Under predation risk, 1,385±133 eggs per aquarium were produced, which was barely half the level of eggs produced under control conditions (2,655±421; *t* = 2.878, df = 4, *p* = 0.045).

**Table 1:** Summary of descriptive statistics of reproduction by *Pomacea canaliculata*. N indicates the number of egg masses.

Variable	N	mean	SE	CV	range
***Clutch size***					
Control	68	117.132	6.517	45.88	34–263
Predation risk	44	94.432	4.741	33.30	22–177
***Oviposition height* (cm)**					
Control	68	17.338	0.535	25.46	5.4–25.5
Predation risk	44	16.698	0.806	32.03	4.9–25.0
***Hatching success* (%)**					
Control	16	71.712	4.656	25.97	31.03–90.98
Predation risk	15	23.503	5.280	87.00	0.00–52.63
***Incubation period* (d)**					
Control	16	13.50	0.387	11.48	12–16
Predation risk	12	14.75	0.429	10.07	12–17

**Table 2:** Summary of nested ANOVA results of reproduction by *Pomacea canaliculata*.

Variable	Factor	df	MS	*F*	*p*
***Clutch size***	Predation risk	1	12022.109	5.545	0.020
	Aquaria (Predation risk)	4	1546.772	0.713	0.585
***Oviposition height***	Predation risk	1	10.157	0.469	0.495
	Aquaria (Predation risk)	4	59.695	2.755	0.032
***Hatching success***	Predation risk	1	18945.623	55.820	0.000
	Aquaria (Predation risk)	4	642.887	1.894	0.143
***Incubation period***	Predation risk	1	11.892	4.593	0.043
	Aquaria (Predation risk)	4	0.821	0.317	0.864

The difference in the oviposition height between the snails in the control and predation risk groups was not significant (Tables 1 and 2). The hatching success was 71.71% in the control group, which was significantly higher than that under predation risk (23.50%, Tables 1 and 2). The egg hatching success of *P*. *canaliculata* was found to be highly variable, ranging from 31.03% to 90.98% in the 16 egg masses that were randomly selected from the control group (Table 1). By contrast, the highest hatching success under predation risk was just over 50%, and 20% of the egg masses did not hatch at all. The *P*. *canaliculata* incubation period did differ significantly between the control and predation risk groups (Tables 1 and 2). The statistical analysis did not include the three unhatched egg masses in the predation risk group.

**Fig 3:**
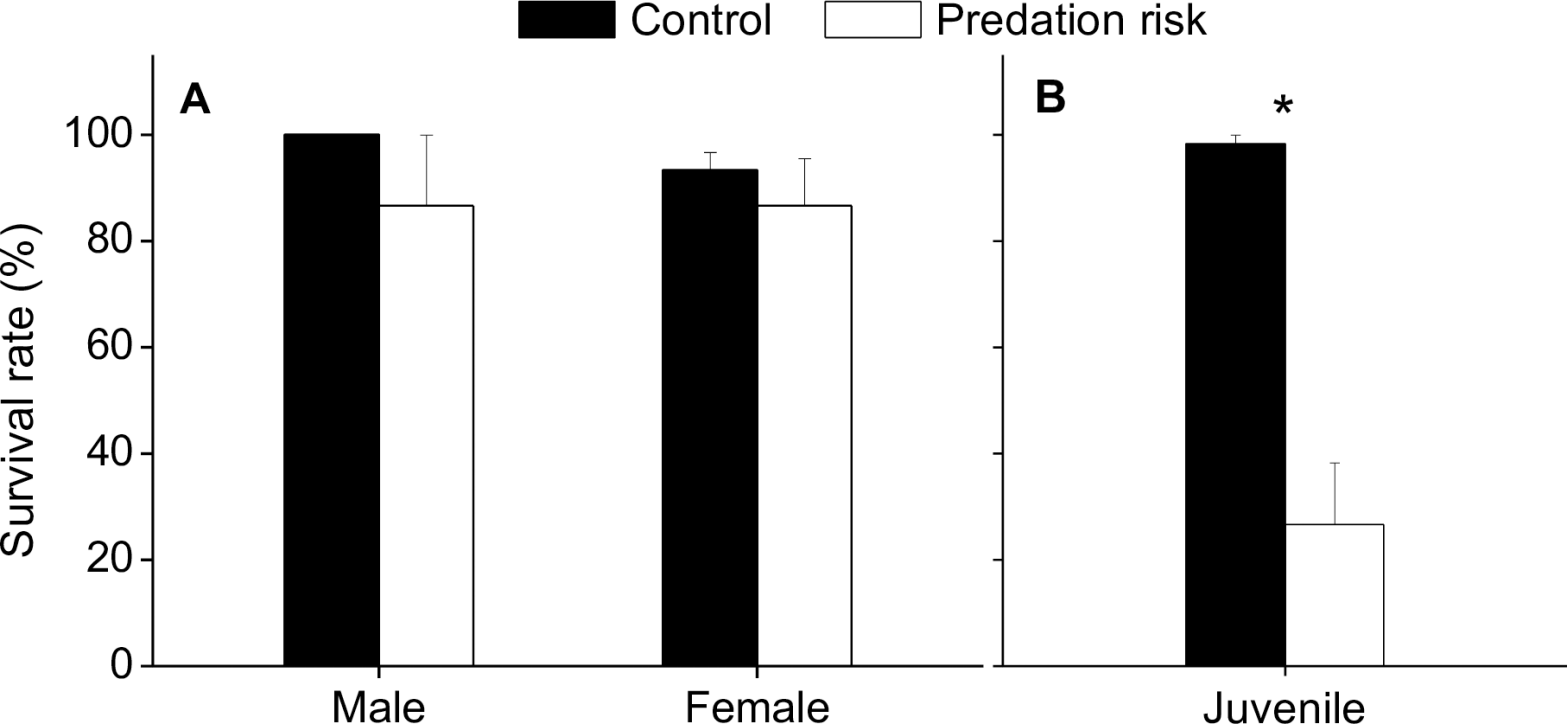
**Survival Rates of Adult (A) and Juvenile (B) *Pomacea canaliculata* under Predation Risk.** The asterisks indicate that the survival rate of *P*. *canaliculata* was significantly different between the control and predation risk groups. * *p*<0.05. Error bars represent standard errors of the mean.

For adult *P*. *canaliculata*, all of the males and 93.33% of the females survived in the control group, whereas only 86.67% of the males and females under predation risk were alive at the end of the experiment. However, no statistically significant differences in the survival rate of either male or female *P*. *canaliculata* were observed between the groups (Male: *Z* = -1.000, *p* = 0.700, Mann-Whitney U test; Female: *t* = 0.707, df = 4, *p* = 0.519, Fig 3A). In stark contrast, a lethal effect of predation risk was observed in juvenile snails, with only 26.67% of the juveniles surviving in the presence of turtles; this survival rate was far below the 98.33% survival rate observed in the control group (*t* = 6.081, df = 4, *p* = 0.004, Fig 3B).

The weights of the individual male (control: 4.04±0.07 g, predation risk: 4.16±0.10 g; *t* = 1.028, df = 4, *p* = 0.362) and female (control: 4.34±0.16 g, predation risk: 4.32±0.12 g; *t* = 0.100, df = 4, *p* = 0.925) adult *P*. *canaliculata* in each group were similar at the start of the experiment, but the growth of the snails was inhibited under the influence of the turtles. The weight of the male snails increased by 57.76% in the control group and was nearly 1.5 times higher than that of those under predation risk, although the difference was not significant (Table 3). However, females under predation risk exhibited significantly lower increases in weight compared with the controls (Table 3). Because juvenile snails (control: 0.22±0.00 g, predation risk: 0.23±0.00 g, experiment initiation time; *t* = 0.543, df = 4, *p* = 0.616) experience a period of rapid growth, their increases in weight in the control group were greater than those in adults (Table 3). However, in the surviving 26.67% of juveniles under predation risk, their growth was strongly inhibited (Table 3). The food intake per *P*. *canaliculata* juvenile during the whole experiment was significantly lower (*t* = 5.004, df = 4, *p* = 0.007) in the predation risk group (1.27±0.44 g) than in the control group (3.94±0.30 g).

**Table 3:** Summary of descriptive statistics and independent sample *t*-test results for the growth of adult and juvenile *Pomacea canaliculata*.

*Weight growth rate* (%)		N	mean	SE	CV	range	df	*t*	*p*
Adult male	Control	3	57.764	8.457	25.36	41.11–68.66	4	1.221	0.289
	Predation risk	3	39.733	12.102	52.76	16.03–55.83			
Adult female	Control	3	59.316	3.688	10.77	52.51–65.19	4	5.345	0.006
	Predation risk	3	25.832	5.064	33.95	18.63–35.60			
Juvenile	Control	3	167.770	15.536	16.04	149.14–198.62	4	8.629	0.001
	Predation risk	3	27.148	4.918	31.38	19.93–36.55			

## Discussion

Our study revealed negative effects of the perceived risk of predation by turtles on the fitness components of an invasive snail. Predation risk affected the fecundity of females, hatching success of eggs, and female growth of adult *P*. *canaliculata*. The negative effects of predation risk on survival and growth were stronger in juveniles than in adults.

In China, the turtle *T*. *scripta elegans* lives primarily in rivers and lakes, and is rarely found in rice fields [37], whereas *P*. *canaliculata* is mainly distributed in shallow waters such as paddies and canals [38]. In addition, the North American turtle and the South American apple snail have no co-evolutionary history in their native ranges nor have the test snails used in the experiments coexisted with any turtle species at the collection site before our research. Therefore, the response of the snail to predation risk by *T*. *scripta elegans* may be quite unspecific [39]. The avoidance behaviors of *P*. *canaliculata* in response to predation risk by the Asiatic turtle *Chinemys reevesii* also support this hypothesis [24–26,29]. Predation risk is likely perceived by the snails in the form of chemicals compounds released by the injured or eaten snails [40,41]. This evidence indicates that the effects found here may be common to other subaquatic vertebrate that are predators of this snail in the invaded range.

Compared with controls, egg laying by *P*. *canaliculata* under predation risk was faster at the beginning of the experiment. Nearly half of the adult snails, especially the females [29], buried themselves in sediment for several hours after perceiving the predation risk. Nonetheless, this did not prevent the females in our study from trying to lay as many eggs as possible despite the risk of predation. Another freshwater snail, *Biomphalaria glabrata*, also showed an increase in egg laying soon after exposure to a trematode parasite without parasitization [42]. Increased egg laying is likely a strategy for the snails to maximize reproduction success when encountering critical threats, and increased early reproductive output by an iteroparous species may be associated with future decreases in the parents’ reproduction [42]. But this compensation response should be advantageous for *P*. *canaliculata*, which have a low probability of long adult life under predator threat [42].

Indeed, the number of egg masses laid by per female *P*. *canaliculata* under predation risk was exceeded by that in the controls in the second half of the experiment; by the end of the experiment the number of egg masses under predation risk was approximately 2/3 of that of the control. Suppressed oviposition, even to the point of a cessation of egg laying, has been observed in other snails under predation risk [43,44]. Generally, the growth and reproductive activities of these snails are irreconcilable with predator avoidance [22].

Furthermore, the clutch size decreased by approximately 20% under predation risk compared with that of the control group. Combining the number of females and egg masses they laid, a significant difference was observed in the total number of eggs laid between the groups; a similar phenomenon has been observed for other freshwater snails [45,46]. Prey show increased allocation of resources to predator avoidance, inevitably resulting in a decreased reproductive output [45]. In addition, the insufficient food intake as a result of behavioral defense under predation risk can also lead to restricted growth and smaller-sized individuals, which could eventually affect the fecundity of *P*. *canaliculata* females, as fecundity is proportional to their size [47].

In the present study, the hatching success of *P*. *canaliculata* eggs in the control group was 71.63%, which was close to the hatching success observed in a survey conducted in Kumamoto City, Japan [48]. However, *P*. *canaliculata* under predation risk showed a marked decline in egg hatching success to just over 20%. Ever-present predation risk has a major adverse effect on normal copulation in some snails [44]. Copulation in *P*. *canaliculata* averages approximately 10 h, and sperm transfer begins two hours after penis-sheath intromission and is related to copulation duration [49]. Perhaps predation risk leads to shorter copulations, resulting in a higher proportion of eggs being unfertilized. In a previous study [27], the proportion of hatched eggs under carp predation risk was not observed to change, which was likely because the adult snails were too large to be influenced by the carp [27] and were thus affected to a lesser extent. In addition, the hatching success of single egg masses was found to be highly variable in several studies [20,50] and perhaps this variability blurred the effects of carp.

In the current study, the oviposition height of *P*. *canaliculata* was not observed to change in the presence of predators, which was likely because the snails are less vulnerable to predation once out of water. The incubation period was prolonged by nearly a day and a half for *P*. *canaliculata* under predation risk (unhatched egg masses in the predation risk group were not considered), which has also been reported for the marine snail *Nucella lamellosa* [51] and some other taxa [52,53]. Theoretically, individuals need to maximize the trade-off between survival and growth, and a delay in hatching under predation risk indicates that in the presence of predators their offspring might be more secure during pre-hatching stages than during post-hatching stages [51]. That is exactly the case for *P*. *canaliculata*, for which the eggs are secured outside the water, which prevents the eggs from becoming prey for freshwater predators. Moreover, the warning coloration of eggs and their antinutritive, antidigestive and neurotoxic properties allow them to successfully avoid potential predators [54]. After hatching, the newborn snails can presumably be captured when they fall into the water; thus, delaying hatching might increase offspring survival.

According to our investigation, the survival of adult *P*. *canaliculata* was not affected by predation risk, which is a common observation in juveniles and adults of other prey species [2,55]. However, for some prey, physiological stress in the presence of predators leads to increased vulnerability to other lethal factors, leading to death [56]. For example, the survival of juvenile *Concholepas conchololepas* snails decreased under predation risk [57], and *Leucorrhinia intacta* dragonfly larvae exhibited high death rates after exposure to caged predators [56]. Even predation risk produced a cross-generational lethal effect on offspring of the freshwater snail *Physa acuta* [10]. In the present study, the lethal effect on *P*. *canaliculata* under predation risk was likely linked to the individual size because most of the deaths were among juveniles with shell heights of 5–15 mm. This result can be partly explained by the fact that juvenile *P*. *canaliculata* are more sensitive to predation risk than adults [23,39], a phenomenon that has been observed in some other species [58,59]. In addition, the sizes of juveniles were similar to those of most of the snails used as turtle food in this study, and previous research has shown that juvenile *P*. *canaliculata* respond more strongly to extracts of conspecifics with similar size than to those of conspecifics with different size [23].

Some lethal or sublethal factors, including insufficient energy intake [57], stress hormones [60], predator feces and secretions may affect the survival of the snails under predation risk. These possibilities require further study. In the particular case of *P*. *canaliculata*, we propose two possible explanations for the increased mortality. The reduction in feeding time due to predation risk is probably responsible for reduced growth [22,26], but may also have had lethal effects in those snails whose levels of stored reserves were already low [61]. These snails probably succumbed to the combined effect of reduced food intake and increased energy expenditure during burrowing. The additional time that the snails under predation risk remained buried may have reduced their O_2_ intake due to the impossibility to reach the water surface with siphon to breathe air, and this in turn may have built up an O_2_-debt with deleterious effects [62].

The present study is the first to report a lethal effect of predation risk on *P*. *canaliculata*, even though the predator avoidance behaviors of this snail have been documented in many studies. The duration of predation risk might be an important factor, as our study lasted 25 days; this study time far exceeded the lengths of other studies, which lasted less than one day because the behavioral responses of *P*. *canaliculata* to predation risk can occur within several hours [23,24,26,29,39].

Defense against predators is often associated with slowed growth in prey species [63]. In this study, the growth rates of adult female and juvenile *P*. *canaliculata* decreased under predation risk. The potential causes likely included a reduction in feeding time associated with the burrowing activity to avoid predators [22,26], as the food intake of juvenile *P*. *canaliculata* under predation risk dropt to 32% of that in the control group. Moreover, the energy expenditure on morphological and immune defenses under predation risk might also cause the growth arrests observed in *P*. *canaliculata* (unpublished data) and other snails [64–66]. The degree of growth inhibition under the risk of predation was greater in adult female snails than in males, which is likely because males show weaker responses to predation risk [29], allowing them to spend more time eating. In addition, females invest more energy into reproduction in order to ensure more offspring. This phenomenon embodies a trade-off between growth and reproduction under inadequate feeding conditions caused by predation risk.

Even though the alarm responses and avoidance behavior of *P*. *canaliculata* [22,26,29] may result in low predation rates in rice fields, it seems plausible that the indirect effects of infochemicals from predators as well as eaten or injured snails may reduce the population growth and spread of the snails through the combined effects on growth, fecundity, hatching success and juvenile survival. However, the effect of the turtle *T*. *scripta elegans* on *P*. *canaliculata* still deserves further consideration in the context of complicated ecosystems, whether the turtle could slow the snail invasion, through predation and the related effects reported here, or accelerate it, by reducing species richness in local communities. Moreover, the presence of the turtle or other predators would inevitably lead to predation risk for *P*. *canaliculata*. The effects of predation risk on prey population dynamics should be taken into full account [6], as these effects are at least half as strong as direct consumption [60]. The predation risk significantly sustains a relatively stable predator-prey dynamics, and its influence is likely to rise with an increasing number of prey [67]. Combined with the multiple effects of predation risk in this study, the risk effects should be included in works that evaluate the performance of predators in controlling the numbers of the invasive snail *P*. *canaliculata*.

## Supporting information

S1 Data Minimal dataset. (XLSX)
